# Injuries of the Skull—their Relation to Medical Evidence, with Reports of Cases, and Remarks upon the Employment of the Trephine

**Published:** 1874-08

**Authors:** C. C. F. Gay

**Affiliations:** Surgeon of the Buffalo General Hospital


BUFFALO

### Medical and Surgical Journal.

VOL XIV. AUGUST, 1874. No. 1.

### Original Communications.

:o:

ART. I.—*Injuries of the Skull—their relation to Medical evidence, with reports of cases, and remarks upon the employment of the Trephine.* By C. C. F. GAY, M. D. Surgeon of the Buffalo General Hospital.

Injuries of the head are so often associated with crime or criminal intent, or at least, their infliction so often becomes a subject of legal investigation, that Surgeons as experts, are called into court to testify how an injury is produced, by what kind of missile or instrument, the amount of danger caused and the probable results to be expected.

The amount of damages to be paid by individuals or corporations or the degree of punishment—whether by imprisonment or death, largely depends upon the testimony of the Surgeon.

But a few days since, the enquiry was made by the able District Attorney, Mr. Williams, whether or not a blow upon the head which did not fracture the skull might be considered as dangerous, or even more dangerous than another blow that fractured the skull. To which I replied affirmatively. He said a case had occurred in court during his term of office, wherein endeavor was made to elicit this fact from medical witnesses, but that there was conflict of opinion.

One of the purposes of the writer in preparing this article, is to examine this question, in order to ascertain if there be any valid reason why medical opinions should not be uniform, and testimony devoid of discrepancy.

The fact, elicited by the question propounded by the District Attorney, is probably well established, still there may be vacillation and uncertainty in the minds of some; if so, I hope to give additional evidence of the fact, by report of cases of injury to the skull, selected from among several recorded in my note-book. The report of the cases themselves it is believed will not be wholly barren of interest.

Case 1.—*Compound fracture of parietal bone.*—Thomas Manning, aged 30 years, admitted to Hospital on April 1st, 1873, was at work excavating earth, when the stone foundation of a building caved in upon him, causing compound fracture of the cranium. I saw the patient soon after the injury was inflicted. He has scalp wound extending from forehead over the vertex to occipital bone down behind the ear, almost entirely denuding the right cranial hemisphere. The parietal bone is fractured through its entire vertical diameter, the fracture runing obliquely downwards in the direction of the posterior inferior angle and depressed quite one quarter of an inch, and three inches or more in extent. Symptoms of compression present. There is no consciousness nor sensibility. Pupils contracted, pulse 30 per minute and intermitting.

Assisted by Drs. Van Peyma and Slacer, and Mr. Bartow I removed a section of bone with the trephine. By the opening thus made I was able, with the use of the handle of strong forceps to elevate the bone. To accomplish this, much strength in the instrument was required and force of muscle necessary to be employed on account of over-riding of the bones.

By a canting motion of the elevator the bone was made to fly back to its normal position with a snapping sound which was audible in the most remote part of the ward.

The elevation of bone was perfect throughout the entire extent of the fracture. Anæsthetics were unnecessary, and were not used.

Almost simultaneous with the elevation of depressed bone, the patient became sensible and conscious. His pulse went up to 60

beats per minute, all symptoms of compression disappeared at once. The scalp wound was dressed, and an anodyne ordered; on April 2d, the patient promised well, on the 3d was worse, his pulse ran up to 140 per minute, and on the afternoon of the 4th he died. I have recorded in my note-book that he died from shock or cerebral contusion or concussion.

CASE 2.—*Compound Comminuted Fracture of Temporal Bone and Tibia.*—Francis McKevett, aged 18 years, fell April 24th, 1873, from the mast of a vessel, distance one hundred feet. The fall was twice broken, the first landing place was upon the cross-tree, a distance of forty feet. He was brought to Hospital in the evening. I first saw him at 10 A. M., next morning.

He has compound comminuted fracture of right tibia near the knee, and there is also compound comminuted fracture of the right temporal bone, with depression, but there are no symptoms of compression. Pulse 120 per minute, pupils normal and consciousness unimpaired. Assisted by Drs. Barnes, Van Peyma and Rolph, I applied the trephine and elevated all depressed bone, and removed several spiculæ of bone which were driven into the brain. It became necessary in removing all the loose fragments of bone, to remove a good share of the temporal bone itself.

Ether was administered, and anæsthesia maintained until the tibia was dressed. Water dressings were order to the head, and an anodyne given. Pulse after operation 120 per minute. April 28th, right leg, and extending a considerable distance upon the thigh, is becoming gangrenous, pulse 140. He is conscious and perspires very much.

30th. Died at 2 o'clock this morning.

Post-mortem at 11 A. M.

Found the leg gangrenous, fracture of the leg oblique, extending well up toward the knee joint. The wound of the head presented no unhealthy appearance.

CASE 3.—*Injuries caused by falling* 40 *or* 50 *feet.—Cardiac displacement without a Fracture or Contusion.*—Lying in close proximity in the Surgical Ward to case 2, was John Krohn, aged 22 years, of full habit, who had fallen on April 10th, 1873, a distance of 40 or 50 feet from the arch of a Propeller, without either fracturing any bones or causing the least contusion anywhere upon his person, yet causing a most unusual and remarkable "shaking up" of a vital organ, namely, displacement of the heart, which was not discovered until a day or two after his entrance to the Hospital. At this time his pulse was 42 per minute with no cerebral symptoms. The apex beat was found near the ensiform cartilage, three inches on a horizontal line below and four inches on a vertical line to the right of the left nipple.

Rest in the recumbent posture was ordered and maintained. On June 12th, he left the Hospital, feeling pretty well. His heart gradually from week to week had moved toward—until it had nearly or quite assumed—its normal position.

Epistaxis occurred twice during his residence in Hospital.

CASE 4.—*Compound Comminuted Fracture of Parietal Bone.*—A lad 11 years of age was struck upon the head by a falling brick from a building in process of construction.

The brick fell probably a distance of 24 feet, causing compound comminuted fracture with depression of left parietal bone. The bone was depressed one-eighth of an inch. I was requested by Dr. Wetmore, to see this case with him in consultation. There were no signs present of compression except unusual dilitation of pupils, and depressed pulse, still the extent of the depression seemed to warrant surgical interference. After the doctor had used the trephine, it was found that the bone could not be elevated by all the force we could use, therefore with the use of gnawing-forceps all of the depressed bone was removed, exposing the meninges of the brain to the extent of one and a half inch in length, by one half inch in breadth. The recovery was uninterrupted. In ten days the boy was up and walking about well. Chloroform was used.

CASE 5.—*Necrosis of the Skull, Operation for Trephining.*—Mr. B. German, aged 40 years, has necrosis of the skull, cannot ascertain that he ever had syphilis, but he admits that he had gonorrhœa twenty years since. His scalp has been inflamed, and suppuration has continued for several months. May 22d, 1871, administered chloroform, cut down upon the bone and find it roughened and necrosed at the posterior superior angle of the parietal bone. The trephine and the gnawing-forceps were employed, by which all the necrosed bone was removed, leaving an opening two inches in diameter.

Water dressings were employed, on the 27th erysipelas supervened upon the face.

June 5th, erysipilas disappeared and the wound was closed. On July 31st, was discharged well.

CASE 6.—*Compound Comminuted Fracture of Arm, and a supposed fracture and depression of Skull.*—Emma Golhe, German, aged 13 years, was brought to General Hospital on the evening of May 11th, 1874. She was exhumed from the ruins of the McArthur building, and taken to Crowley's morgue. From the time the building fell until the body was recovered, nearly an hour and a half intervened. She was supposed to be dead, but soon exhibiting signs of life, was removed to Hospital. It was ascertained that she had compound comminuted fracture of left humerus near the shoulder. There was found depression upon the frontal bone one and a half inches in length by three-fourths of an inch in width, with sharp abrupt margins. There were found other injuries upon the person, but these were slight, such as contusions and one laceration of gastrocnemius muscle.

During her ride to Hospital, she became semi-conscious, pupils were dilated and reaction was established.

Ether was administered, when, assisted by Drs. Boardman, Bartow and Boysen, I amputated the arm, and decided to wait until morning for symptoms of compression before exploring the head injuries. Several physicians saw this patient while she was lying apparently lifeless at the morgue, all of them concurred in opinion that there was fracture of the cranium. The depression was deep with sharp edges, but upon lifting a flap, which as a rule I could not be justified on authority in doing, for the purpose of making diagnosis, I discovered there was neither fracture nor depressed bone. The deformity was such as to lead almost any one into error of diagnosis, and in case of this character where several competent physicians were deceived, it ought to furnish an exception to the rule, never to cut for the mere purpose of establishing a diagnosis. On the next day the patient's pulse ran to 170 and 180, convulsions supervened, she gradually became unconscious, and died on the morning of the 14th, having lived three days after receiving the injury.

These cases, separately, are interesting and instructive, but when grouped together furnish a text for remarks, having a broader scope than would be afforded, had not the cases been brought together in this connection. Cases first and second necessitated employment of the trephine. The first case was one of severe concussion and was fatal. The other case would not have been fatal if there had been no complex injury. Cases fourth and fifth required employment of the trephine, but the cause which necessitated its use, was not of a nature to produce more than slight shock in one case, while the other was unattended by shock, and both patients recovered.

Case fourth which I have reported, a lad eleven years of age, recovered without an untoward symptom. His age was in his favor, but the case could not be included in the class of those reported by Dr. Hamilton, in the Buffalo Medical Journal, Nov., 1846. He had passed the period of infancy and childhood, when non-interference is advised. With the presure of bone upon the brain removed, he is exempt from future risk of epileptoid convulsions and death from this cause.

Many years since I witnessed an operation for trephining, by Dr. Pancost, of Philadelphia, upon an epileptic boy. The injury had been inflicted nine years before, by a fall upon a curbstone. The lad had enjoyed good health for several years after the accident, when convulsions supervened, said to be epileptic in character recurring at intervals more and more frequent. Dr. P., decided to perforate the frontal bone, removing therefrom a spicula of bone which projected from the inner plate. Only for this delay to operate, for which the doctor was not responsible, the lad would have been spared the convulsions and his life saved.

Case third is reported along with the other cases, as showing the effect upon the system—when the force of a blow is not broken by giving way of bones in any part of the body—of general concussion. It could not be ascertained how or where this patient struck, whether upon his head or side or feet. But the presumption is, that he did not strike upon the head, for it would hardly be possible for one to fall the distance of 40 feet, striking upon the head without producing, if not fracture, at least a wound or contusion upon the scalp or cerebral concussion. I regret that I am unable to state the termination of the case. Adopting the views of Erichsen, enunciated in his able work on concussion of the spine in Rail Road injuries, I can readily prognose a fatal termination within from two to three years.

In the two fatal cases, the fracture was in one case simple, in the other comminuted. In the former the compression was profound. In the latter the symptoms of compression were scarcely appreciable. A section of the parietal bone in one case was flattened down upon the meninges of the brain to considerable extent. A smaller section of bone in the other case was comminuted, the fragments of bone depressed, and spiculæ driven down upon the brain substance. Why were there profound symptoms of compression in the one and not in the other? The solution of the question may be found in the fact that the cerebral shock is lessened in proportion to the degree and amount of comminution. In this case the fractured leg diminished the cerebro-spinal shock. Erichsen happily illustrates this point by stating, that if a "watch falls to the floor without breaking its crystal, the work of the watch stops and perchance will be permanently injured, but if the crystal breaks in the fall the shock is lessened, so that the watch keeps on running and is not injured." "When a magnet is struck a heavy blow with a hammer, the magnetic force is jarred, shaken, or concussed out of the horse shoe, and the iron has lost its magnetic power," and this is about as good a definition of the term "shock" as can be given at present.

A person, says this author, "who by any of the accidents of civil life meets with an injury by which one of the limbs is fractured or is dislocated, necessarily sustains a very severe shock, but it is the rarest thing possible to find that the spinal cord or the brain has been injuriously influenced by this shock that has been impressed on the body.

It would appear as if the violence of the shock expended itself in the production of the fracture or the dislocation, and that a jar of the more delicate nervous structures is thus avoided."

This question of "shock" or concussion from Railway and other injuries of the nervous system has often come up in the courts, and the testimony of physicians has not always been of a character to boast or be proud of. Discordant testimony has exerted its influence in bringing into bad repute the standing of the profession.

Erichsen has done much by way of establishing principles and harmonizing conflicting views in relation to injuries inflicted by railway accidents, so much so that there need not hereafter as heretofore be such conflict in medical opinion on questions of diagnosis, pathology and prognosis, in injuries such as cause "shock" to the nervous system. I think I am prepared to assert on authority, that comminuted fracture of the cranium is less fatal than simple fractures, and that comminuted or simple fractures are less dangerous than no fracture at all provided the shock, in all cases, is equal.

I am quite positive therefore, should I be called upon to testify as an expert, that I should be obliged under the sanctity of an oath to state my belief, that a blow which did not fracture the skull, being equal in momentum to another blow upon the head causing fracture, either simple, compound or comminuted, would be the more dangerous and fatal even, of the two.

Personal experience and observation, together with the light thrown upon the subject matter by Erichsen's theory of injuries causing cerebral or cerebro-spinal concession, would be the basis of this belief.

In one case, the shock or shaking up, would be so much more intense than in another, that an expert could be able with certainty to prognose a fatal termination within a given period of time, from acute or chronic meningitis or subacute myelitis, or permanent breaking down of the nervous system.

"In the spine, just as in the head," says Erichsen, "it will sometimes be found after death from what appears to be, and in reality is, simple injury of the nervous centres, that the vertebral column in the one case, and the skull in the other, have suffered an amount of injury that was unsuspected during life; and which, though it may not in any way have determined to the fatality of the result, yet affords conclusive evidence of the violence to which the parts have been subjected, and the intensity of the disorganizing shock that they have suffered.

There is, however, this very essential difference between the spine and the head in these respects—that a simple fracture of the cranium may be of no moment except so far as the violence that has occasioned it may have influenced the brain, whilst in the spine the case is not parallel; for as the vertebral column is the centre of support to the body, its influence in this respect will be lost when broken; even though the spinal cord may not have been injured by the edges of the fractured vertebræ, but simply violently and fatally concussed by the same force that broke the spine itself."

Holmes cites Drummond's case as an example of extensive destruction of the vault of the skull, originating in a blow upon the head without fracture or scalp wound. The destruction of the vault being caused by inflammation starting in the diplœ.

It is conceded by the best authority that comminuted fractures are limited in their consequences to the seat of injury, and that compound fractures are much more frequently limited to the seat of injury than in cases of simple fracture. Says Holmes: "Out of 56 cases of simple fracture this strict limitation existed only in one single instance."

These views, held by the highest surgical authority, have a direct bearing upon the question propounded by the district attorney and furnishes an affirmative answer.

Of these two most recent and very able writers and authors—namely, Erichsen and Holmes, it may be affirmed that the former errs by placing too much emphasis upon the term "concussion," as limited to the brain and spinal cord, while the latter, being more conservative, strikes the happy medium when he says: "Thus all the great constituents of the nervous system—brain, spinal cord, and sympathetic system—are included in the common risk of the catastrophe. And the account would be incomplete, if the influence of mental shock—that of fright and of witnessing appalling spectacles—were neglected."

In this connection it is not irrelevant to observe that much obloquy has been thrown upon the operation of trephning which it does not deserve, since so many fatal cases occur after this operative procedure. But much of the discredit belongs not to the operation, but rather to the causes which make the operation imperative and necessary. The causes may have been so severe in character, producing such instant and severe injury—not by way of lesion to the soft or hard parts—but to the nervous centres that death, which is inevitable, is neither deferred nor hastened by the operation of trephining. Yet there are risks in the future from omission to elevate depressed bone, the certainty of the occurrence of which no man may be able to determine, which justify the employment of the trephine.

It must of necessity be an injury—severe in character—that suggests the employment of the trephine. Its use can add but little if any to the shock already inflicted. Trephining is one of the simplest operations in surgery. But little pain is caused—an anæsthetic is scarcely called for, but little time is consumed or blood lost, and in these severe injuries of the head, ablation of a section of bone may be readily effected, whether the fracture be comminuted or not by the use of the gnawing-forceps without use of trephinei, therefore I cannot but think that more immediate and remote danger to patient results from non-use than from use of this instrument or its congener, the gnawing-forceps. Well established rules are laid down by authors regulating the employment of the trephine. These rules and their exceptions are nowhere better defined and more succinctly stated than they are in Hamilton's Principles and Practice of Surgery.

I am quite well convinced, however, that no rule can be so inflexibly established that there shall be no departure therefrom· Exceptions will arise and may be made to all rules that are now or may hereafter be laid down and apparently established. Yet while a surgeon, without reputation, may be obliged to follow these rules for his own professional safety, a surgeon with reputation, will occasionally find it expedient and necessary for the safety of his patient to depart from them. I am unable to bring myself to the belief that in case of fracture with depression and without compression in the adult, that good surgery, as a rule, requires me to wait for the development of symptoms. Promptness in decision and action, at least in exceptional cases, and the elevation of depressed bone should be aimed at, when the full duty of the surgeon has been done and the consequences may be left to take care of themselves.

The Divine Architect has so wonderfully constructed the human cranium as to enable it to resist great violence. Any one who will, may demonstrate upon the cadaver the immense power necessaary to be brought into requisition, adequate to a fracture of the cranial bones. Whosoever, I think, makes the trial, will be convinced that the impact of a force that fractures the bones of the skull must almost of necessity so contuse the brain substance or inflict injury upon the investing membranes, or cause extravisation and blood clot with or without fracture as to produce, with the attendant shock, inevitable death, whether the trephine be employed or not, but should doubt arise in the mind of the surgeon as to the propriety of its use, in legal parlance, give the patient the benefit of the doubt for, perchance, benefit may accrue to him by prompt decision and action and future risks avoided.

At all events, but little if any more is added to the so called shock of the injury by the employment of the trephine. The surgeon indeed cannot quite feel at rest and certain that his whole obligation to his patient is discharged if he leaves any portion of the cranial vault depressed. Still authority seems to have set at rest the question of operative, interference or non-interference, to swerve from which would be dangerous to the young surgeon if it should not prove to be so to his patient.

A little more warrant for an occasional deviation from the rule is all I should be willing to insist upon, and this warrant I suspect will, in due time, be granted.

